# Supplementary material for: Multi‐dataset identification of innovative feature genes and molecular mechanisms in keratoconus
Source: J Cell Mol Med. 2024 Sep 19;28(18):e70079. doi: 10.1111/jcmm.70079 (PMC11412914; doi:10.1111/jcmm.70079)
Supplement: Supplementary file 2 — Material S2. [file JCMM-28-e70079-s002.zip › 7.GSEA analysis/6.SNORA74B/KEGG_EPITHELIAL_CELL_SIGNALING_IN_HELICOBACTER_PYLORI_INFECTION.html]

Details for gene set KEGG\_EPITHELIAL\_CELL\_SIGNALING\_IN\_HELICOBACTER\_PYLORI\_INFECTION[GSEA]

|  || Dataset | SNORA74B.group.cls#h\_versus\_l |
| Phenotype | group.cls#h\_versus\_l |
| Upregulated in class | l |
| GeneSet | KEGG\_EPITHELIAL\_CELL\_SIGNALING\_IN\_HELICOBACTER\_PYLORI\_INFECTION |
| Enrichment Score (ES) | -0.48347902 |
| Normalized Enrichment Score (NES) | -1.6796274 |
| Nominal p-value | 0.020283977 |
| FDR q-value | 0.2310328 |
| FWER p-Value | 0.443 |
Table: GSEA Results Summary

  

Fig 1: Enrichment plot: KEGG\_EPITHELIAL\_CELL\_SIGNALING\_IN\_HELICOBACTER\_PYLORI\_INFECTION      
 Profile of the Running ES Score & Positions of GeneSet Members on the Rank Ordered List

  

| SYMBOL | TITLE | RANK IN GENE LIST | RANK METRIC SCORE | RUNNING ES | CORE ENRICHMENT || 1 | ATP6V0D2 | na | 3824 | 0.266 | -0.0821 | No |
| 2 | CXCR2 | na | 5077 | 0.223 | -0.0946 | No |
| 3 | ATP6V0A4 | na | 6445 | 0.178 | -0.1151 | No |
| 4 | PLCG2 | na | 8239 | 0.131 | -0.1530 | No |
| 5 | MAPK11 | na | 8296 | 0.129 | -0.1409 | No |
| 6 | IKBKB | na | 8763 | 0.119 | -0.1417 | No |
| 7 | PLCG1 | na | 9672 | 0.099 | -0.1574 | No |
| 8 | CASP3 | na | 9789 | 0.097 | -0.1504 | No |
| 9 | MAPK9 | na | 10427 | 0.085 | -0.1598 | No |
| 10 | TJP1 | na | 11082 | 0.073 | -0.1709 | No |
| 11 | MAPK13 | na | 11114 | 0.073 | -0.1641 | No |
| 12 | MAPK12 | na | 11855 | 0.062 | -0.1788 | No |
| 13 | ATP6V0D1 | na | 11942 | 0.061 | -0.1749 | No |
| 14 | MAPK14 | na | 12827 | 0.049 | -0.1952 | No |
| 15 | PTPN11 | na | 13916 | 0.037 | -0.2226 | No |
| 16 | CSK | na | 14025 | 0.035 | -0.2220 | No |
| 17 | ATP6V0B | na | 14649 | 0.028 | -0.2369 | No |
| 18 | ATP6V1G3 | na | 14960 | 0.025 | -0.2433 | No |
| 19 | GIT1 | na | 14994 | 0.024 | -0.2416 | No |
| 20 | MAPK10 | na | 14996 | 0.024 | -0.2390 | No |
| 21 | MAP2K4 | na | 16268 | 0.012 | -0.2745 | No |
| 22 | ATP6V1D | na | 16292 | 0.012 | -0.2739 | No |
| 23 | CHUK | na | 16402 | 0.010 | -0.2759 | No |
| 24 | ATP6V1B2 | na | 16733 | 0.007 | -0.2847 | No |
| 25 | ATP6AP1 | na | 16940 | 0.005 | -0.2901 | No |
| 26 | F11R | na | 17230 | 0.002 | -0.2982 | No |
| 27 | ATP6V0E1 | na | 17370 | 0.001 | -0.3021 | No |
| 28 | ATP6V0A1 | na | 17685 | -0.002 | -0.3109 | No |
| 29 | ATP6V1F | na | 18118 | -0.007 | -0.3226 | No |
| 30 | ATP6V1C1 | na | 18619 | -0.013 | -0.3356 | No |
| 31 | CDC42 | na | 18770 | -0.015 | -0.3384 | No |
| 32 | ATP6V0C | na | 19442 | -0.022 | -0.3554 | No |
| 33 | EGFR | na | 19596 | -0.024 | -0.3573 | No |
| 34 | RAC1 | na | 19881 | -0.027 | -0.3626 | No |
| 35 | MAPK8 | na | 20414 | -0.033 | -0.3744 | No |
| 36 | IKBKG | na | 20820 | -0.038 | -0.3821 | No |
| 37 | ATP6V1G1 | na | 21282 | -0.043 | -0.3908 | No |
| 38 | ATP6V1A | na | 21423 | -0.045 | -0.3901 | No |
| 39 | NOD1 | na | 22251 | -0.055 | -0.4081 | No |
| 40 | ATP6V1E1 | na | 22282 | -0.055 | -0.4031 | No |
| 41 | HBEGF | na | 22304 | -0.055 | -0.3979 | No |
| 42 | ADAM10 | na | 22979 | -0.065 | -0.4104 | No |
| 43 | ATP6V1C2 | na | 23997 | -0.081 | -0.4312 | No |
| 44 | ADAM17 | na | 24187 | -0.084 | -0.4278 | No |
| 45 | ATP6V1H | na | 24227 | -0.084 | -0.4199 | No |
| 46 | JUN | na | 25317 | -0.104 | -0.4403 | No |
| 47 | ATP6V1B1 | na | 25630 | -0.110 | -0.4377 | No |
| 48 | ATP6V0E2 | na | 26252 | -0.122 | -0.4427 | No |
| 49 | NFKB1 | na | 26534 | -0.128 | -0.4371 | No |
| 50 | ATP6V1E2 | na | 28141 | -0.168 | -0.4656 | Yes |
| 51 | PAK1 | na | 28478 | -0.179 | -0.4563 | Yes |
| 52 | NFKBIA | na | 28570 | -0.182 | -0.4397 | Yes |
| 53 | MET | na | 28809 | -0.189 | -0.4265 | Yes |
| 54 | LYN | na | 29275 | -0.203 | -0.4184 | Yes |
| 55 | RELA | na | 29651 | -0.214 | -0.4065 | Yes |
| 56 | SRC | na | 30227 | -0.234 | -0.3982 | Yes |
| 57 | MAP3K14 | na | 31321 | -0.285 | -0.3995 | Yes |
| 58 | CXCL8 | na | 31337 | -0.285 | -0.3697 | Yes |
| 59 | ATP6V1G2 | na | 32051 | -0.325 | -0.3558 | Yes |
| 60 | CCL5 | na | 32237 | -0.337 | -0.3254 | Yes |
| 61 | IGSF5 | na | 33399 | -0.438 | -0.3124 | Yes |
| 62 | PTPRZ1 | na | 33424 | -0.441 | -0.2663 | Yes |
| 63 | JAM2 | na | 34268 | -0.600 | -0.2270 | Yes |
| 64 | CXCR1 | na | 34414 | -0.656 | -0.1616 | Yes |
| 65 | CXCL1 | na | 34497 | -0.699 | -0.0898 | Yes |
| 66 | JAM3 | na | 34668 | -0.909 | 0.0017 | Yes |
Table: GSEA details [plain text format]

  

Fig 2: KEGG\_EPITHELIAL\_CELL\_SIGNALING\_IN\_HELICOBACTER\_PYLORI\_INFECTION      
 Blue-Pink O' Gram in the Space of the Analyzed GeneSet

  

Fig 3: KEGG\_EPITHELIAL\_CELL\_SIGNALING\_IN\_HELICOBACTER\_PYLORI\_INFECTION: Random ES distribution      
 Gene set null distribution of ES for **KEGG\_EPITHELIAL\_CELL\_SIGNALING\_IN\_HELICOBACTER\_PYLORI\_INFECTION**
